# Serological evidence of *Borrelia turicatae* in Raccoons (*Procyon lotor*) from an endemic focus of tick-borne relapsing fever in Mexico

**DOI:** 10.1371/journal.pntd.0014242

**Published:** 2026-04-22

**Authors:** Jose A. Hernández Martínez, Valeria Leal-Sepúlveda, Alan A. Zavala-Norzagaray, Emilio Rendón-Franco, César P. Ley-Quiñonez, Patricio Pellegrini-Hernández, Job E. Lopez, J. Antonio Ibarra

**Affiliations:** 1 Departamento de Microbiología, Escuela Nacional de Ciencias Biológicas, Instituto Politécnico Nacional, Ciudad de México, México; 2 Instituto Politécnico Nacional, CIIDIR-SIN, Guasave, Sinaloa, México; 3 Departamento de Producción Agrícola y Animal, Universidad Autónoma Metropolitana-Unidad Xochimilco, Ciudad de México, México; 4 Wildlife Conservation Management Unit (Macochín), El Fuerte, Sinaloa, México; 5 Department of Pediatrics, National School of Tropical Medicine, Baylor College of Medicine, Houston, Texas, United States of America; University of Notre Dame, UNITED STATES OF AMERICA

## Abstract

Tick-borne relapsing fever is a neglected and overlooked disease. In Mexico, numerous historical reports document the distribution of *Ornithodoros turicata*, the vector of *Borrelia turicatae*, as well as human cases of infection. However, the enzootic cycle and reservoir hosts in Mexico remain unknown. Here, to detect previous infections with relapsing fever Borreliae in wild fauna a retrospective serological analysis was conducted with serum samples collected from raccoons trapped from 2022 to 2025 in the Navachiste region of Sinaloa, Mexico. Using a species-specific antigen, BipA from *B. turicatae*, and bacterial lysates of this spirochete, we found high exposure among this cohort (30/36 sera, 83.3%). These results indicate the role of raccoon in this area as frequent host of *B. turicatae* and, together with previous findings, suggest a possible endemic focus of tick-borne relapsing fever in northern Mexico. Our findings remark the need for further investigation into the ecoepidemiology of *B. turicatae* in this region.

## Author summary

Despite being originally described in Mexico, tick-borne relapsing fever caused by *Borrelia turicatae* is poorly understood and understudied. Historical and recent findings show that *Ornithodoros turicata* is distributed from central to northwestern Mexico, and the first *B. turicatae* was isolated from ticks in northern Sinaloa, suggesting naturally occurring enzootic cycles. Our study provides serological evidence of raccoons´ high exposure to *B. turicatae* in three sites in northern Sinaloa, Mexico, with varying degrees of human impact. We provide valid evidence to support surveillance and public health attention in these potential emerging endemic foci.

## Introduction

Tick-borne relapsing fever (TBRF) is caused by spirochetes of the genus *Borrelia*, primarily transmitted through the bite of *Ornithodoros* soft ticks [1]. In North America, *Ornithodoros turicata* is the main TBRF vector, found from the southwestern to central United States, with an isolated group in Florida, and extending into northern and central Mexico [2–4]. In Mexico, at least four other *Ornithodoros* species have been reported as potential vectors of TBRF *Borrelia*: *Ornithodoros talaje*, *Ornithodoros dugesi*, *Ornithodoros coriaceus* and *Ornithodoros puertoricensis* [4]. While TBRF spirochetes have been largely overlooked in Mexico, *Borrelia turicatae* and *Borrelia puertoricensis* were isolated from *O. turicata* and *O. puertoricensis*, respectively, confirming their role as competent vectors in Mexico [5,6].

All but one species of TBRF spirochetes is maintained in enzootic cycles yet the wildlife hosts that maintain the pathogens in nature still remains unclear. For instance, exposure to *B. turicatae* has been shown in raccoons and wild canids [7]. This is in part due to the life cycle of the pathogens in the vertebrate host. For example, TBRF spirochetes are blood-borne pathogens and attain upwards of 1 x 10^7^ bacteria per mL of blood [8]. The host generates an antibody response that clears the predominant population of TBRF spirochetes, and they are undetectable in the blood [9]. Antigenic variation causes a new population to emerge in host’s blood, and this dynamic between the host antibody response and antigenic variation can persist for months. One approach to identify a competent vertebrate host requires direct isolation from the blood during a period of spirochetemia. However, sampling wild animals at the appropriate time is largely contingent on chance [10,11].

An alternative approach to identify competent vertebrate hosts is serological surveillance. In a competent host, laboratory studies indicate that if a strain of TBRF spirochetes attains 1x10^4^ bacteria per mL of blood or higher the spirochetes can be acquired by ticks and complete their life cycle [8]. Moreover, at this level of spirochetemia the host generates a detectable antibody response [8], which could be targeted to identify competent hosts. One diagnostic antigen that has been used to identify the species of TBRF spirochete causing infection is recombinant *Borrelia* immunogenic protein A (rBipA) [12–14]. This antigen has been used in serological surveys showing *Borrelia* exposure in human patients from Sonora and Sinaloa, Mexico [15,16], indicating that these regions are endemic for TBRF spirochetes.

Currently, there are no reports regarding wildlife exposure to *B. turicatae* from Mexico detected by immunological means, and providing such evidence will improve the understanding of the eco-epidemiology of this spirochete. In this study, to make an approximation for the exposure of wild fauna to *B. turicatae* in an area where this bacterium has been proven to be present in soft ticks we tested sera from resident raccoons (*Procyon lotor*) to detect anti-*Borrelia* antibodies. Results showed that most of these animals in the Navachiste region of Sinaloa, Mexico, were positive for the species-specific antigen rBipA from *B. turicatae*. This study provides a framework for future surveillance of wildlife in Sinaloa, Mexico.

## Methods

### Ethics statement

All blood samples were collected in accordance with animal welfare guidelines [17], and under the approval of the Secretaría de Medio Ambiente y Recursos Naturales (SEMARNAT) (SGPA/DGVS/0564/21) from the Mexican government. This protocol was approved by our ethics committee at ENCB-IPN (ECO-002–2025).

### Study area

**Fig 1:**
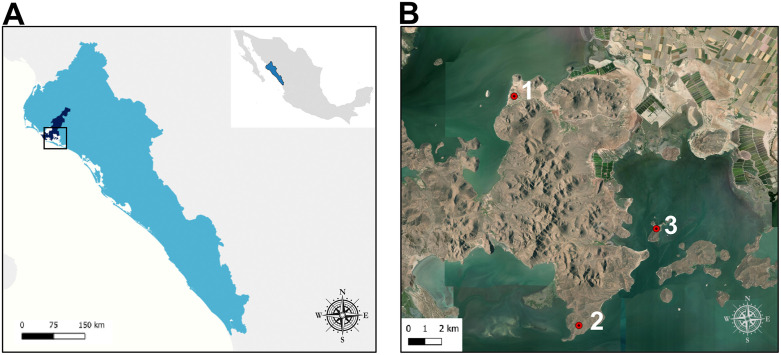
Raccoon capturing and sampling locations in Sinaloa, Mexico. (A) The map shows the state of Sinaloa in light blue, the municipality of Juan José Ríos highlighted in dark blue, and the black outlined square indicates where sampling was conducted and zoomed in (B). The inset in the upper right corner of (A) shows the location of Sinaloa within Mexico. Collection sites (B) including: 1) Lázaro Cárdenas, 2) Sierra de Navachiste, and 3) Isla de Las Chivas. Terrain maps were produced using QGIS v.3.40 with layers accessible from GADM (https://gadm.org/) (A), and USGS EROS (Earth Resources Observatory and Science (EROS) Center) (public domain: https://earthexplorer.usgs.gov/) (B).

Raccoons were captured at three sites (Fig 1) with different levels of human impact in northern Sinaloa, Mexico, for four continuous years (2022–2025) by using convenience sampling: a) Lázaro Cárdenas (LC) (25° 35′ 59″ N, 108° 58′ 3″ W): A small fishing community near the Bahía de Ohuira with approximately 794 inhabitants, half of them from the Indigenous Mayo-Yoreme population; b) Sierra de Navachiste (SN) (25° 27’ 43” N, 108° 55’ 43″ W). A state natural protected area and part of two Ramsar sites (number 1826 and 2025) [18]. The vegetation consists of tropical deciduous forest and mangroves. This area lacks permanent human activity but is surrounded by agricultural and livestock land. In addition to fishing, livestock and agriculture are practiced; and c) Isla de as Chivas (IC) (25° 30′ 55″ N, 108° 54′ 24″ W): A 65-hectare island located south of SN and part of a federal protected area (Área de Protección de Flora y Fauna “Islas del Golfo de California”). It has the same vegetation as SN and lacks permanent human settlements, though it is occasionally used for recreational purposes. All three sites have a transition of semi-dry and hot to BS (h’) to very dry and hot (BW(h’)) [17,18].

### Animal trapping

Captures were conducted between November 2022 and October 2025, using Tomahawk box traps [11]. Traps were placed in areas with signs of animal activity, such as trails or waterholes, and in anthropized areas, near waste disposal sites [19]. Traps were baited with sardines, jam, and canned cat food, set before sunset, and checked shortly after sunrise [20].

Captured animals were sedated with ketamine-xylazine (10 mg/kg) or tiletamine-zolazepam (5 mg/kg) using a pole syringe [21]. Each animal was sexed, aged, and marked with a PIT-tag for individual identification [11]. Animals were evaluated for ticks. Blood samples were collected from the jugular vein, placed in Vacutainer tubes without anticoagulant. After the clothing samples were centrifuged at 1,500 rpm for 15 min. Serum was placed in 1.5 mL vials and stored at 4 °C until arrival at the laboratory, where it was stored at -20 °C until analysis [22].

### Serology

Western blot was performed to assess seroconversion against *B. turicatae* 91E135 [23] whole-cell lysates and purified rBipA of *B. turicatae* as described previously [14,15]. Briefly, lysates from 1 × 10⁷ *B. turicatae* spirochetes and 1 µg of purified rBipA were used. Proteins were separated by SDS-PAGE (1.5 h) and transferred to polyvinylidene difluoride membranes (Millipore, Billerica, MA) using a semi-dry transfer system (Amersham, Inc). Membranes were blocked overnight with Tropix I-Block (Thermo Fisher Scientific, Waltham, MA), then incubated for 1 h at room temperature with serum samples diluted 1:200. The secondary protein was HRP-conjugated protein A/G (Thermo Fisher Scientific, Waltham, MA) at a 1:5,000 dilution. Signal detection was performed using the SuperSignal West Pico Chemiluminescent Substrate Kit (Thermo Fisher) and visualized with a Chemidoc Touch Imaging System (Bio-Rad, Hercules, CA). A sample was considered positive if it contained antibodies reactive to 5 or more proteins in the whole-cell lysate and to the rBipA protein [7,10].

### Statistics

The frequency and the confidence interval 95% Fisher Exact (Clopper-Pearson) were calculated for all samples and for each capture site. All analyses were done with the open software OpenEpi [24].

## Results

### Sample cohort

By performing a convenience sampling protocol, a total trapping effort of 97 trap-nights was conducted (35 per site; except 27 trap-nights for LC), resulting in a capture success rate of 37%. A total of 36 raccoons (*P. lotor*) were captured as follows: 16 from Lázaro Cárdenas, 7 from Sierra de Navachiste, and 13 from Isla de Las Chivas (Table 1). No ticks were found on any of the captured individuals.

**Table 1:** Raccoons captured and their seroreactivity against *B. turicatae* antigens.

Location	No. positive/No. tested	Positive % (CI95%)
Lázaro Cárdenas	14/16	87.5 (61.6-87.8)
Sierra de Navachiste	6/7	85.7 (42.1-99.6)
Isla de Las Chivas	10/13	76.9 (46.1-94.9)

#### Detection of anti-*Borrelia* antibodies.

**Fig 2:**
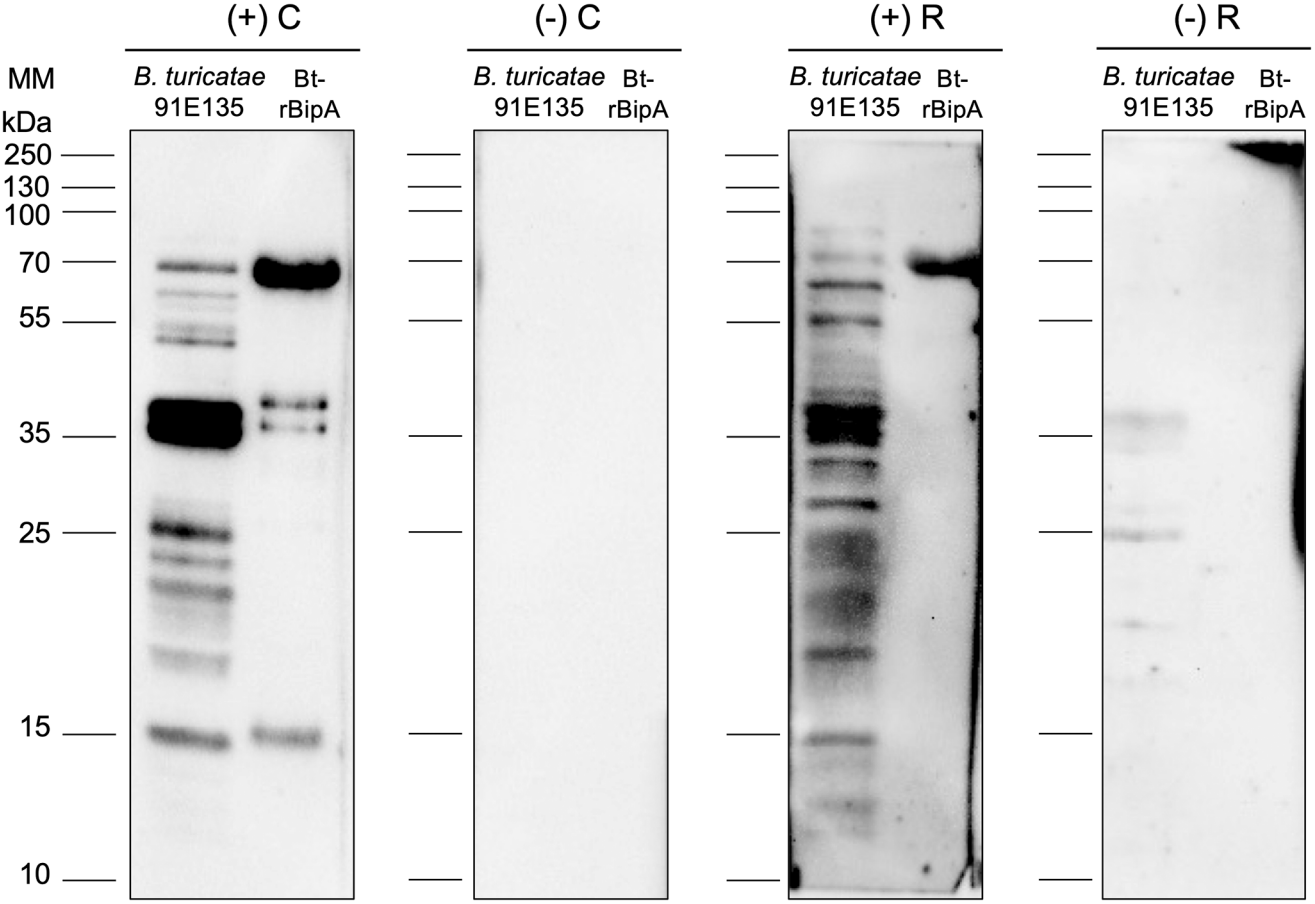
Serological evaluation of raccoons to detect antibodies against rBipA and *B. turicatae* 91E135 bacterial lysate. Serum samples were used to a 1:200 dilution. Serum from a mouse immunized with *B. turicatae* 91E135 was used as a positive control (+C), and preimmune mouse serum as a negative control (–C). Shown are two examples positive (+R) and negative (-R) raccoon serum samples. Molecular masses are shown to the left of each panel.

Serum samples were tested against *B. turicatae* protein extract and rBipA from this same species. Out of the 36 raccoon serum samples, 30 (83.3%, CI95% 67.1-93.6) showed immunoreactivity to *B. turicatae* rBipA and to the whole-protein lysate proteins (Table 1 and Fig 2). Our criteria to identify a positive sample was antibody reactivity to five or more proteins in the whole-cell lysate and to the purified rBipA [7,10]. These results indicate that raccoons in the studied cohort were exposed to *B. turicatae*.

## Discussion

In this study, we initiated an assessment of potential vertebrate hosts of *B. turicatae* in Mexico by detecting exposure of raccoons to relapsing fever Borreliae. Although experimental infections with *Borrelia* spp. have been performed in laboratory animals such as mice, rats, rabbits, guinea pigs, and non-human primates [25,26], little is known about the infection of wild fauna. A few wild mammals, such as opossums, foxes, rodents etc., have also been found naturally infected with *Borrelia* sp., suggesting their role as vertebrate reservoirs [10,11,27–31]. However, in this part of the Americas, records of *B. turicatae* in wildlife are scarce [32]; expanding knowledge on potential vertebrate hosts is therefore essential to begin understanding the enzootic cycle of this spirochete in Mexico. We selected the Navachiste region in Sinaloa because we already demonstrated that *O. turicata* in the neighbor region of Camayeca harbors *B. turicatae* [5].

Addionally, prior work suggests that raccoons may serve as competent hosts for *B. turicatae.* For instance, serological evidence of exposure to *B. turicatae* in raccoons was shown to be 8.0% in Texas, suggesting that this mammal may serve as competent hosts for this spirochete [7]. Moreover, studies on the bloodmeal sources of *O. turicata* have found high feeding frequencies on raccoons (*P. lotor*), opossums (*Didelphis virginiana*), and armadillos (*Dasypus novemcinctus*) [33,34]. As mentioned above, anti-*Borrelia* antibodies have been shown for raccoons [7], but not for opossums and armadillos. Overall, data on potential *Borrelia* reservoirs in Latin America remain scarce.

Our results reveal high exposure of the tested raccoons to *B. turicatae* across all three study sites at the Navachiste region in Sinaloa. Across all three study sites, this exposure rate of raccoons to *B. turicatae* indicate the need for enhanced surveillance and warrants further studies with a bigger population of raccoons and other vertebrates in this area. This finding carries several implications because raccoons might be competent hosts in this region, indicating the presence of infected *O. turicata* ticks. Ongoing investigations include the capture of soft ticks in these three regions to detect and isolate *B. turicatae*. In addition, given the synanthropic behavior of raccoons [35,36], potential zoonotic events may occur highlighting the relevance of surveilling wild fauna in natural protected areas, as well as populated areas, as ecological disturbance can impact overall ecosystem health. Raccoons can move across large areas [37], potentially facilitating the spread of pathogens to new locations, provided a competent tick vector is present; however, additional studies are required to corroborate this hypothesis and to demonstrate that ticks can acquire the spirochete from infected blood. As in other zoonotic diseases, this underscores the need for integrated “One Health” surveillance strategies in endemic regions [38] and shows that wild fauna such as raccoons can be used as sentinels [39].

Our results support findings from a previous study in which we explored the ecology of *B. turicatae* in Texas and show that wild canids and raccoons were seropositive for *B. turicatae*-specific antibodies [7]. Before the present report, exposure of wild fauna in Mexico to *Borrelia* sp. has been shown by ELISA to bacterial extracts or by PCR [32], however, these methods do not allow for the specific detection of anti-*Borrelia* antibodies and may produce false negatives due to the relapsing nature of the infection [16]. The use of specific antigenic recombinant proteins allows for the accurate detection of pathogen exposure and the precise identification of the responsible agents.

In summary, here we report, for the first time, serological evidence of exposure to *B. turicatae* in raccoons in northern Mexico. Such levels of exposure are not uncommon in TBRF-endemic areas. For instance, members of the family Sciuridae have shown seropositivity ranging from 40% to 90% for *Borrelia hermsii* in TBRF endemic regions of the United States [11,31]. Our findings highlight the need for further investigation into the ecology and transmission dynamics of *B. turicatae* in this region. Specifically, efforts are needed to obtain and analyze a bigger sample size to understand the ecology and dispersal of *B. turicatae* among raccoons, but also multiple wild fauna and domestic animals (cattle, dogs, cats, goats, etc.) should be included. Comparative studies on the genetic diversity of *Borrelia* strains between island and mainland populations are also warranted. These findings align with the recent isolation of *B. turicatae* approximately 50 km northwest of the sampling area [5], suggesting that this region may represent an emerging endemic focus of *B. turicatae*. We suggest continuing the search for other vertebrates as competent hosts of *B. turicatae* and potential reservoirs, and the use of raccoons as serological sentinel species for TBRF, contributing to wildlife disease surveillance programs in Mexico [39,40]. Moreover, to determine whether humans have been exposed to *B. turicatae* which will allow to stablish a program for the diagnosis of TBRF in this region.
